# Activation of Coronary Arteriolar PKCβ2 Impairs Endothelial NO-Mediated Vasodilation: Role of JNK/Rho Kinase Signaling and Xanthine Oxidase Activation

**DOI:** 10.3390/ijms22189763

**Published:** 2021-09-09

**Authors:** Naris Thengchaisri, Travis W. Hein, Yi Ren, Lih Kuo

**Affiliations:** 1 Department of Medical Physiology, Cardiovascular Research Institute, College of Medicine, Texas A&M University Health Science Center, Bryan, TX 77807, USA; ajnaris@yahoo.com (N.T.); thein@tamu.edu (T.W.H.); yren@tamu.edu (Y.R.); 2 Department of Companion Animal Clinical Sciences, Faculty of Veterinary Medicine, Kasetsart University, Bangkok 10900, Thailand

**Keywords:** oxidative stress, coronary disease, phorbol esters, nitric oxide, vasodilation, vasoconstriction, superoxide

## Abstract

Protein kinase C (PKC) activation can evoke vasoconstriction and contribute to coronary disease. However, it is unclear whether PKC activation, without activating the contractile machinery, can lead to coronary arteriolar dysfunction. The vasoconstriction induced by the PKC activator phorbol 12,13-dibutyrate (PDBu) was examined in isolated porcine coronary arterioles. The PDBu-evoked vasoconstriction was sensitive to a broad-spectrum PKC inhibitor but not affected by inhibiting PKCβ2 or Rho kinase. After exposure of the vessels to a sub-vasomotor concentration of PDBu (1 nmol/L, 60 min), the endothelium-dependent nitric oxide (NO)-mediated dilations in response to serotonin and adenosine were compromised but the dilation induced by the NO donor sodium nitroprusside was unaltered. PDBu elevated superoxide production, which was blocked by the superoxide scavenger Tempol. The impaired NO-mediated vasodilations were reversed by Tempol or inhibition of PKCβ2, xanthine oxidase, c-Jun N-terminal kinase (JNK) and Rho kinase but were not affected by a hydrogen peroxide scavenger or inhibitors of NAD(P)H oxidase and p38 kinase. The PKCβ2 protein was detected in the arteriolar wall and co-localized with endothelial NO synthase. In conclusion, activation of PKCβ2 appears to compromise NO-mediated vasodilation via Rho kinase-mediated JNK signaling and superoxide production from xanthine oxidase, independent of the activation of the smooth muscle contractile machinery.

## 1. Introduction

Protein kinase C (PKC) is a critical intracellular signaling molecule that orchestrates various vascular functions, including gene expression [1], cell differentiation [2] and proliferation [1], angiogenesis [3], permeability [4], vesicle trafficking [5], and vasoconstriction [6,7,8,9,10,11]. Cumulative evidence suggests a close relationship between PKC activation and production of reactive oxygen species (ROS) in vascular cells subjected to high pressure or hyperglycemic challenge [12]. Therefore, activation of PKC links to oxidative stress-associated vascular complications and blood flow dysregulation during disease development, including diabetes [13,14], ischemia-reperfusion injury [15], dyslipidemia [16], atherosclerosis [1], and hypertension [12]. Moreover, inhibition of PKC has been shown to attenuate vascular superoxide production in various forms of cardiovascular stress in animals [14,17,18] and humans [14,19]. Interestingly, recent studies suggested a role of mitogen-activated protein kinases (MAPKs) or Rho kinase in superoxide production from coronary microvessels subjected to inflammatory insults [20,21,22] or harvested from animals with cardiovascular diseases [21,23,24]. However, it remains unclear whether direct activation of PKC signaling in the healthy vasculature can cause MAPK/Rho kinase activation and excessive superoxide production, with consequent vasomotor dysfunction.

It is well established that the vascular smooth muscle activity can be modulated by vasoactive substances released from the endothelium [25]. Numerous studies have shown that micromolar concentrations of phorbol 12,13-dibutyrate (PDBu), a direct PKC activator, evoke a sustained vasoconstriction in various tissues [7,8,9,10,11,26]. Interestingly, the endothelium-dependent vasodilation can be blunted by PDBu due to inhibition of synthesis/release and the action of nitric oxide (NO) [8]. These studies indicate that PKC activation not only elicits vasoconstriction but also compromises endothelial NO function. Therefore, the endothelial dysfunction is likely to accentuate the vasoconstriction following PKC activation. Alternatively, the vasoconstriction evoked by PKC can potentially mask endothelium-dependent vasorelaxation. Furthermore, the finding that the synthesis/release of endothelial vasodilators is influenced by the contractile activity of the underlying vascular smooth muscle cells [27,28] adds another complexity to vasomotor regulation by PKC. Interestingly, the elevated smooth muscle Ca^2+^ during contraction can feedforward to the endothelium to evoke vasodilation [29]. This aforementioned crosstalk between smooth muscle and endothelial cells can confound interpretation of the endothelial effect of PKC in vasomotor regulation if the smooth muscle contractile machinery is also activated by the PKC. Therefore, the direct impact of PKC activation on endothelial NO function and the involved signaling molecules in association with oxidative stress remain unclear. 

Herein, we directly addressed the impact of PKC activation by PDBu on endothelium-dependent NO-mediated dilation of isolated porcine coronary arterioles using a sub-vasomotor concentration of PDBu. The specific PKC isoform and the role of p38 kinase, c-Jun N-terminal kinase (JNK), and Rho kinase in oxidase activation and superoxide production were also determined in these microvessels, which are known to be responsible for coronary blood flow regulation [25,30].

## 2. Results

### 2.1. Vasomotor Effect of PDBu and PKC Involvement

**Figure 1:**
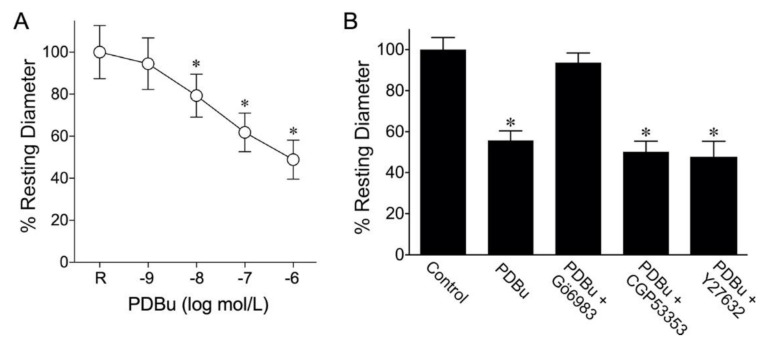
PDBu elicited concentration-dependent constriction of porcine coronary arterioles. (**A**) The low concentration of PDBu (1 nmol/L) had no effect on vascular tone, but higher concentrations (≥10 nmol/L) caused significant vasoconstrictions (*n* = 6). * *p* < 0.05 vs. resting diameter (R), one-way repeated measures ANOVA with Dunnett’s multiple comparison test. (**B**) PDBu-induced vasoconstriction (PDBu 0.1 µmol/L, *n* = 5) was prevented by the pan-PKC inhibitor Gö6983 (1 µmol/L, *n* = 5) but was not affected by the PKCβ2 inhibitor CGP53353 (0.3 µmol/L, *n* = 5) or Rho kinase inhibitor Y27632 (0.1 µmol/L, *n* = 5). * *p* < 0.05 vs. the control, one-way ANOVA with Dunnett’s multiple comparison test.

To evaluate the vasomotor effect of PDBu, the isolated coronary arterioles were exposed to cumulative concentrations of PDBu and the responses were recorded. Coronary arterioles (96 ± 5 µm maximum diameter; *n* = 10) developed a stable basal tone (67 ± 2% of maximal diameter) and constricted in response to PDBu in a concentration-dependent manner (Figure 1A). PDBu at a 1 nmol/L concentration did not cause vasoconstriction but at 1 µmol/L it elicited a 50% reduction in the resting diameter (Figure 1A). The vasoconstriction caused by PDBu (0.1 µmol/L) was abolished by a pan-PKC inhibitor Gö6983 (1 µmol/L, *n* = 5) but was not affected by a PKCβ2 inhibitor CGP53353 (0.3 µmol/L, *n* = 5) or Rho kinase inhibitor Y27632 (0.1 µmol/L, *n* = 5) (Figure 1B). 

**Figure 2:**
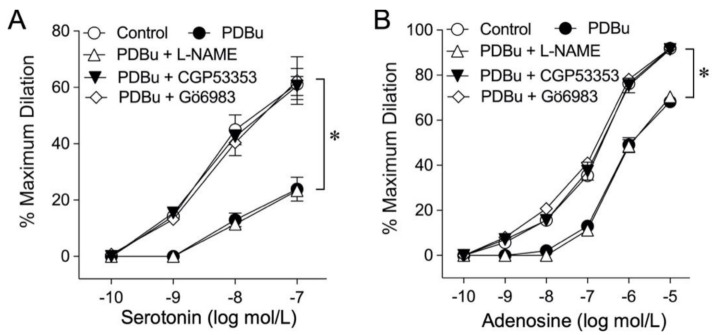
Impact of PKC activation on NO-mediated vasodilations to serotonin and adenosine. Vasodilations in response to serotonin (**A**) and adenosine (**B**) were inhibited by pre-treating the vessels with 1 nmol/L PDBu for 60 min (*n* = 6). The pan-PKC inhibitor Gö6983 (1 µmol/L, *n* = 5) and PKCβ2 inhibitor CGP53353 (0.3 µmol/L, *n* = 5) prevented the PDBu-induced impairment of endothelium-dependent vasodilations. NOS inhibitor L-NAME (10 µmol/L, *n* = 4) did not alter the inhibitory effect of PDBu. * *p* < 0.05 vs. the control, two-way repeated measures ANOVA with Tukey’s multiple comparison test.

In another series of experiments, the endothelium-dependent, NO-mediated vasodilations in response to serotonin (0.1 nmol/L to 0.1 µmol/L) and adenosine (0.1 nmol/L to 10 µmol/L) were examined before and after treating the vessel with a sub-threshold concentration (1 nmol/L) of PDBu for 60 min. PDBu did not significantly alter the resting diameter of the vessels but inhibited the arteriolar dilations in response to serotonin (Figure 2A) and adenosine (Figure 2B). Addition of NO synthase (NOS) inhibitor L-NAME (10 µmol/L) did not affect the inhibitory effect of PDBu on vasodilations (Figure 2). Co-incubation of PDBu with a pan-PKC inhibitor Gö6983 or with PKCβ2 inhibitor CGP53353 prevented the inhibitory effect of PDBu (Figure 2). PDBu (1 nmol/L) had no effect on the vasodilation elicited by the NO donor sodium nitroprusside (Figure S1). 

### 2.2. Role of ROS, NAD(P)H Oxidase, and Xanthine Oxidase in the PDBu-Induced Superoxide Production and Vascular Dysfunction

**Figure 3:**
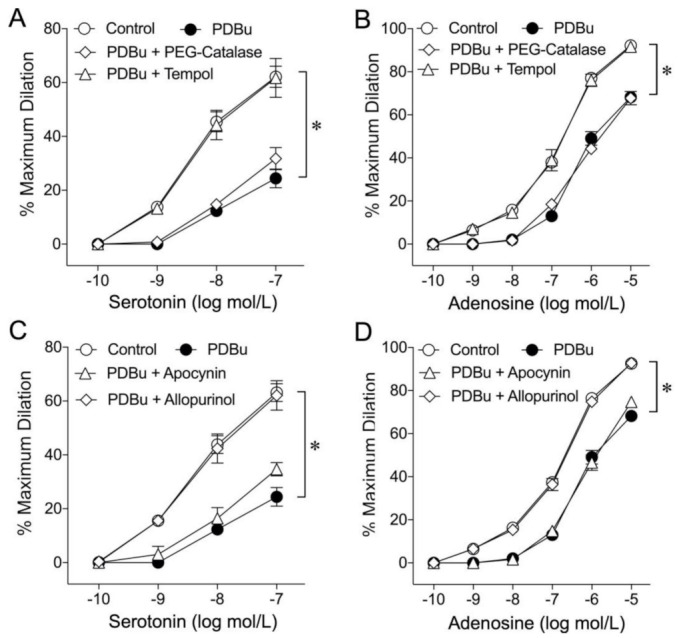
Role of oxidative stress in the adverse effect of PKC activation. The inhibitory effect of PDBu on vasodilations in response to serotonin (**A**) and adenosine (**B**) was prevented by pre-treating the vessels with the superoxide scavenger Tempol (1 mmol/L, *n* = 5) but not by the cell permeable H_2_O_2_ scavenger PEG-catalase (500 U/mL, *n* = 5). The inhibitory effect of PDBu on vasodilations induced by serotonin (**C**) and adenosine (**D**) was prevented in vessels treated with the xanthine oxidase inhibitor allopurinol (100 µmol/L, *n* = 5). However, the NAD(P)H oxidase inhibitor apocynin (100 µmol/L, *n* = 5) had no effect on PDBu-induced vascular impairment in response to serotonin (**C**) and adenosine (**D**). * *p* < 0.05 vs. the control, two-way repeated measures ANOVA with Tukey’s multiple comparison test.

**Figure 4:**
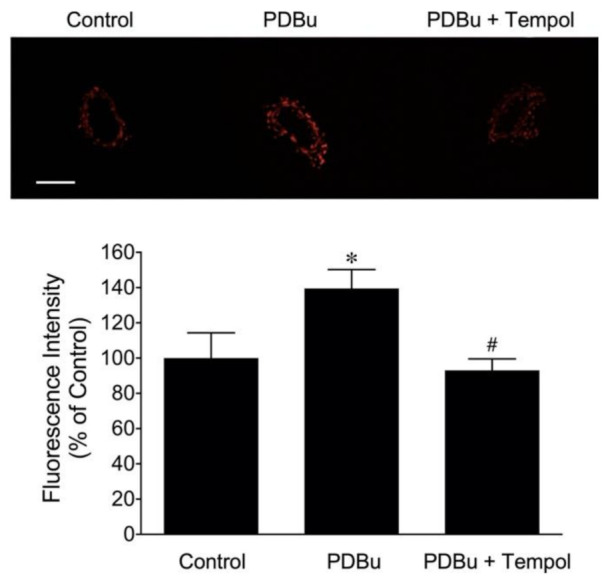
Detection of superoxide production in porcine coronary arterioles. Dihydroethidium (DHE) imaging of superoxide production in coronary arterioles after incubation of the vessels with PDBu (1 nmol/L, 60 min). PDBu markedly increased the superoxide level in the vascular wall, which was significantly reduced by co-incubation with the superoxide scavenger Tempol (*n* = 3). The result of the quantitative analysis of the DHE fluorescence signals is shown. Scale bar represents 100 µm. The data represent three independent experiments. * *p* < 0.05 vs. the control, and # *p* < 0.05 vs. PDBu treatment, Student’s *t*-test.

To determine whether ROS is involved in the attenuation of endothelium-dependent NO mediated vasodilation, vessels were treated with PDBu (1 nmol/L) in the presence of either the superoxide scavenger Tempol (1 mmol/L) or H_2_O_2_ scavenger PEG-catalase (500 U/mL). Pretreating the vessels with Tempol completely prevented the adverse effects of PDBu on vasodilations in response to serotonin (Figure 3A) and adenosine (Figure 3B). However, the cell-permeable H_2_O_2_ scavenger PEG-catalase had no effect on the inhibitory action of PDBu (Figure 3A,B). To determine whether NAD(P)H oxidase or xanthine oxidase contributes to the adverse effect of PDBu, vessels were treated with PDBu in combination with inhibitor of NAD(P)H oxidase (apocynin, 100 µmol/L) or of xanthine oxidase (allopurinol, 10 µmol/L). In the presence of allopurinol, but not apocynin, the vasodilations to serotonin (Figure 3C) and adenosine (Figure 3D) were preserved. The vessels treated with PDBu significantly elevated the superoxide production in a manner sensitive to Tempol (Figure 4).

### 2.3. Expression of NOS and PKCβ2 in Coronary Arterioles

**Figure 5:**
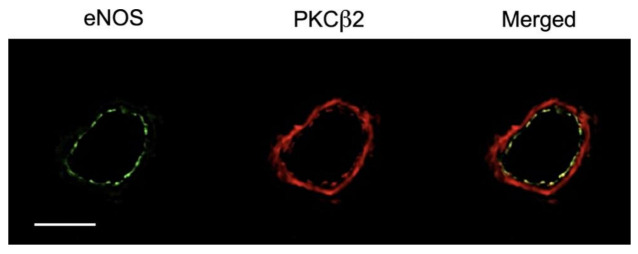
Immunohistochemical detection of endothelial NOS (eNOS) and PKCβ2 in porcine coronary arterioles. The expression of PKCβ2 (red) was found in smooth muscle and endothelial cells and co-localized with eNOS (green) in the endothelial layer (three independent experiments). Scale bar represents 50 µm.

To investigate the vascular expressions of endothelial NOS (eNOS) and PKCβ2, immunohistochemical detection of these two proteins was performed in isolated porcine coronary arterioles with a size comparable to that used for functional studies. The expression of PKCβ2 was found in both smooth muscle and endothelial cells and co-localized with eNOS in the endothelial layer (Figure 5).

### 2.4. Role of JNK, p38 Kinase, and Rho Kinase in the Inhibitory Effect of PDBu

**Figure 6:**
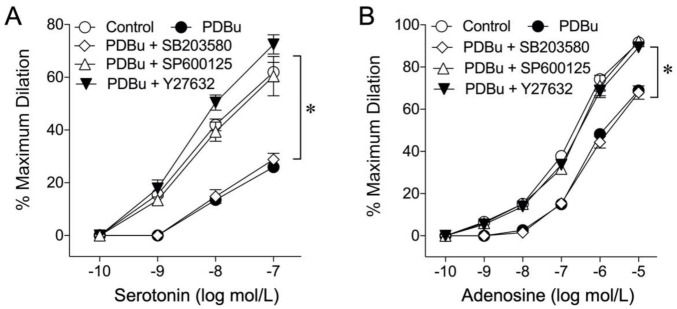
Role of kinases in the adverse effect of PKC activation. In the presence of the JNK inhibitor SP600125 (5 µmol/L, *n* = 5) or Rho kinase inhibitor Y-27632 (0.1 µmol/L, *n* = 5), but not p38 kinase inhibitor SB203580 (0.1 µmol/L, *n* = 5), the adverse effect of PDBu on vasodilations in response to serotonin (**A**) and adenosine (**B**) was prevented. * *p* < 0.05 vs. the control, two-way repeated measures ANOVA with Tukey’s multiple comparison test.

To investigate the involvement of JNK, p38 kinase, and Rho kinase in the adverse effect of PDBu, the vasodilator responses were examined after treating the vessels with PDBu (1 nmol/L) in the presence of the respective kinase inhibitor. The inhibitory effect of PDBu on vasodilations induced by serotonin (Figure 6A) and adenosine (Figure 6B) was prevented in the presence of the JNK inhibitor (SP600125, 5 µmol/L) or Rho kinase inhibitor (Y-27632, 0.1 µmol/L). However, the p38 kinase inhibitor (SB203580, 0.1 µmol/L) had no effect on the action of PDBu (Figure 6).

## 3. Discussion

PKC plays a central role in signal transduction for vasoconstriction and tissue inflammation and is thought to be involved in development of diabetes and atherosclerosis [1,31] as well as other coronary diseases associated with endothelial dysfunction [32,33], which may underlie coronary vasospasm [34]. In the present study, activation of PKC by a sub-vasomotor concentration of PDBu elicits endothelial dysfunction by inhibiting endothelium-dependent, NO-mediated vasodilations (adenosine and serotonin) via elevated oxidative stress. The activation of xanthine oxidase by PKCβ2, linking to JNK and Rho kinase signalings, contributes to overt superoxide production and vascular dysfunction. 

Although PKC was first identified as a Ca^2+^-activated phospholipid-dependent protein kinase, various isoforms were subsequently discovered and categorized into three subfamilies according to their structures and activators: conventional/classic (cPKCs), novel (nPKCs), and atypical (aPKCs). Activation of four cPKCs isoforms (α, β1, β2, and γ) requires Ca^2+^ and diacylglycerol (DAG), whereas the four nPKCs isoforms (δ, ϵ, η, and θ) are activated by DAG alone [35]. The activation of aPKCs (ι/λ, and ζ) is dependent upon phosphatidic acid and phosphatidylserine but not DAG or Ca^2+^ [36]. In cPKC signaling, binding of Ca^2+^ increases the affinity of cPKCs for membrane phosphatidylserine and promotes cPKCs binding to DAG at the cell membrane. Binding to DAG releases cPKC from autoinhibition, exposing the active site of cPKC for target phosphorylation. The pharmacological DAG analog, PDBu, acts in a similar manner to DAG to evoke PKC activation. It has been shown that micromolar concentrations of PDBu can cause vasoconstriction in various tissues via PKC activation [7,8,9,10,11,26]. Interestingly, in the swine model of coronary balloon injury, the PKC-mediated constrictions of coronary arteries to PDBu (1 nmol/kg with estimated maximal concentration of 0.1 μmol/L) or autacoids are significantly augmented in a Ca^2+^-dependent manner, in which the responses are blocked by the non-selective PKC inhibitor staurosporine [37]. This in vivo study suggests the involvement of cPKC activation in mediating the hyper-constriction, or vasospasm, of coronary arteries in the diseased state. However, the signaling mechanisms underlying the abnormal vascular reactivity remain unclear. It is possible that the diminished counteraction from the endothelial-released vasodilators due to balloon injury also contribute to or superimpose onto the enhanced vasoconstriction during PKC activation. It is unclear whether PKC activation, without evoking vasoconstriction, can cause endothelial dysfunction in healthy vessels. 

In the present study, PDBu caused constriction of the coronary arterioles sensitive to the broad-spectrum PKC inhibitor (Gö6983) toward cPKCs, but not to the selective PKCβ2 inhibitor CGP53353 or Rho kinase inhibitor Y-27632 (Figure 1). These results excluded the involvement of PKCβ2 and Rho kinase in vasoconstriction to cPKC activation. In many cases, PKC has contraction-promoting effects, such as phosphorylation of plasma membrane channels/pumps to increase intracellular Ca^2+^ or phosphorylation of proteins that regulate cross-bridge cycling or Ca^2+^ sensitivity of contractile filaments in smooth muscle [26,38]. Similarly, Rho kinase can modulate vasomotor function by increasing Ca^2+^ sensitivity through inhibition of the myosin light chain phosphatase [26,38]. Interestingly, in the study of aortic vasomotor activity, the contraction elicited by PKC activation is sensitive to Rho kinase inhibitor, suggesting the interaction of these two signaling pathways [9,10]. The explanation for these inconsistent findings is unclear, but our results agree with previous studies in the microvasculature that Rho kinase plays no role in PKC-induced vasoconstriction [39]. It appears that the link of PKC activation to Rho kinase signaling for vasomotor control is mainly in conduit blood vessels [9,10] in a manner consistent with an in vivo study showing the activation of PKC-mediated Rho kinase signaling in development of vasospasm in large coronary arteries under inflammatory insult [34]. On the other hand, the constriction of small resistance arteries to PKC activation seems to be independent of Rho kinase, suggesting the segmental disparity of vasculature in the involvement of Rho kinase signaling downstream from PKC activation [39]. It is worth noting that the concentrations of CGP53353 and Y-27638 used in the present study are adequate to inhibit PKCβ2 [40,41,42] and Rho kinase [43,44,45,46,47], respectively, as reported in various types of tissues.

The balance between smooth muscle tone and endothelial function in the microcirculation is essential for maintaining adequate tissue perfusion [25]. The endothelium plays an important role in blood flow regulation by controlling smooth muscle activity via released NO [30,48]. On the other hand, the increased smooth muscle tone can suppress endothelium-dependent NO-mediated dilation [49]. We have previously reported that both serotonin [50] and adenosine [51] cause endothelium-dependent NO-mediated dilation of porcine coronary arterioles through different signaling mechanisms. Interestingly, treating coronary arterioles with a sub-vasomotor concentration of PDBu (1 nmol/L) blunted vasodilations to both serotonin and adenosine (Figure 2). It appears that this effect is specifically related to the reduction of NO availability from the endothelium rather than the alteration of smooth muscle responsiveness to NO because inhibition of NOS in these PDBu-treated vessels did not further reduce the vasodilations (Figure 2) and the response of the vessels to the NO donor sodium nitroprusside remained unaltered (Figure S1). Moreover, our data suggest that PKCβ2 is involved in the development of endothelial dysfunction because application of its specific inhibitor prevented the adverse effect of PDBu. This finding is consistent with a previous study showing that PKCβ2 activation links to the impairment of flow-induced vasodilation in a microvascular network by suppression of NO release from the endothelium [52]. Interestingly, inhibition of PKCβ2 preserves endothelium-dependent vasodilation [53] and reverses endothelial barrier dysfunction [54] in experimental models with hyperglycemia and diabetes, which are known to cause endothelial NO deficiency [14,23,52,55]. It should be noted that PKCβ2 is expressed abundantly in the coronary arteriolar wall, including endothelial cells, and is co-localized with eNOS (Figure 5). In this regard, the observed endothelial dysfunction with compromised vasodilation in the present study is associated with activation of PKCβ2 without coupling to vasoconstriction. It appears that the induction of endothelial NO deficiency, compared to the initiation of smooth muscle contraction, is more sensitive to PKC activation in coronary arterioles. 

Many cardiovascular diseases are associated with PKC activation [1,13,14,15], leading to elevated superoxide [56,57,58] or hydrogen peroxide [59,60] production. In the present study, the impaired NO-mediated vasodilation by a sub-vasomotor concentration of PDBu was prevented by the membrane-permeable superoxide scavenger Tempol, but not by the hydrogen peroxide scavenger PEG-catalase (Figure 3A,B), suggesting the specific contribution of superoxide to the adverse effect of PKC activation. This context is supported by the detection of an elevated superoxide level in the vascular wall after exposure to a sub-vasomotor concentration of PDBu (Figure 4). Tempol did not alter the resting vascular tone or vasodilator responses to serotonin, adenosine, and sodium nitroprusside [20] but preserved the endothelium-dependent vasodilations (Figure 3A,B) and reduced superoxide production in the PDBu-treated vascular wall (Figure 4). These data, in corroboration with the assessment of vascular function discussed above, support the role of elevated superoxide production in vascular dysfunction following PKC activation. 

Activation of superoxide-producing enzymes, NAD(P)H oxidase and/or xanthine oxidase, has been shown to link to PKC activation [11,14,56,58]. Our results showed that allopurinol, but not apocynin, prevented the adverse effect of PDBu, indicating that xanthine oxidase was responsible for the superoxide production following PKC activation in coronary arterioles (Figure 3C,D). Interestingly, it has been reported that NAD(P)H oxidase is a source of oxidative stress in cultured vascular cells subjected to PKC activation by phorbol myristate acetate (PMA) or high glucose [61]. It is unclear whether the differences in the experimental model (cell culture vs. intact vessel) or the use of different PKC activators (PMA and high glucose vs. PDBu) between this and our study have contributed to the inconsistent results. Nevertheless, it has been shown that, in contrast to PDBu, superoxide production induced by PMA is not always associated with a change in cytosolic PKC activity [62]. It also cannot be excluded that the high concentration of PMA (0.5 µM) used in the above cell culture study might also have triggered other signaling pathways for NAD(P)H oxidase activation. Our results are consistent with a previous study showing that the PKC-dependent xanthine oxidase-mediated superoxide production contributes to coronary endothelial dysfunction and NO deficiency in the heart perfused with inflammatory vasoconstrictor endothelin-1 or angiotensin-II [63]. Xanthine oxidase is also a major source of superoxide to compromise endothelial function and NO bioavailability in porcine [21] and murine [64] coronary arterioles subjected to inflammatory insults and in hypertensive/hypercholesterolemic patients exhibiting impaired coronary endothelium-dependent vasodilation [65]. Therefore, the mechanisms behind the observed functional inhibition by PDBu in the present study involved activation of PKCβ2 and generation of superoxide, a direct NO scavenger, by xanthine oxidase. 

Previous studies have shown that activation of various protein kinases, including Rho kinase and stress-activated protein kinases (p38 MAPK and JNK), can contribute to vasoconstrictor signaling [24,66] or oxidative stress-associated vasomotor dysfunction [20,21,22,67]. Although the nanomolar concentration of PDBu employed in the present study was not sufficient to activate vasomotor activity, the responsible signaling kinase for PKC-induced endothelial dysfunction remained to be determined. In the present study, application of SP600125, but not SB203580, preserved vasodilations to serotonin and adenosine (Figure 6), indicating the involvement of JNK, rather than p38 kinase, in PKCβ2-mediated endothelial dysfunction. Interestingly, the impaired endothelium-dependent vasodilation by oxidative stress in coronary [22] and retinal [43] arterioles after C-reactive protein exposure is mediated by the activation of p38 kinase-dependent NAD(P)H oxidase. However, the role of PKC was not investigated in previous C-reactive protein studies. It appears that different stress environment triggers different signaling pathways, leading to vascular dysfunction. Nevertheless, our current study agrees with the study showing activation of JNK signaling and subsequent production of superoxide via xanthine oxidase under TNFα-mediated inflammatory insult [21]. It is worth noting that TNFα can activate PKCβ2 and promotes oxidative stress in cultured human vascular endothelial cells [68], although the role of JNK in this in vitro study is unclear. Our present studies suggest that activation of PKCβ2, without confounding influence from the vasoconstrictor activity, can elicit oxidative stress via JNK-dependent xanthine oxidase activation and consequently compromise endothelial function in coronary arterioles. 

**Figure 7:**
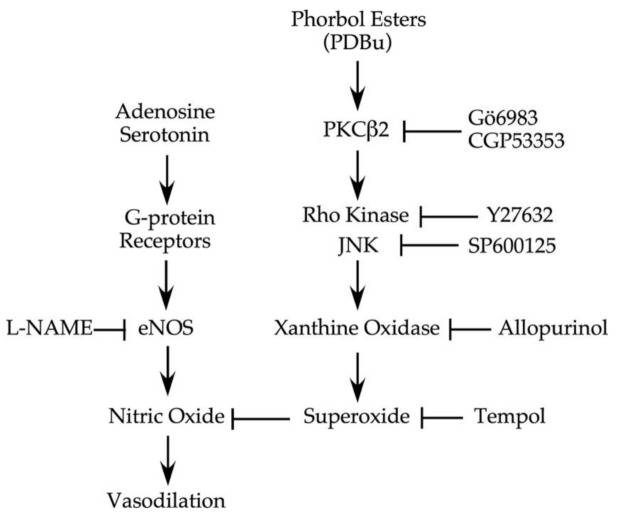
The diagram summarizes the pathways involved in endothelial dysfunction elicited by PDBu. Both serotonin and adenosine activate their G-protein coupled receptors and elicit endothelium-dependent nitric oxide (NO)-mediated vasodilation via endothelial NO synthase (eNOS). Activation of PKC by a nanomolar concentration of PDBu has a minimal effect on vascular tone but compromises NO-mediated vasodilations in response to adenosine and serotonin due to increased superoxide production. The signaling events through Rho kinase and JNK act in concert following PKCβ2 activation to promote superoxide production from xanthine oxidase and consequently lead to NO deficiency. The inhibitors used in the present study to probe the involved signaling molecules are indicated.

Rho kinase activation has been shown to promote superoxide production and endothelial dysfunction in the porcine retinal [43,45] and coronary arterioles [24]. Inhibition of Rho kinase increases vascular NO production [69,70] and improves endothelial function in various vascular diseases, in which augmented vasoconstriction is commonly observed [24,65,70,71]. In the present study, we found that a Rho kinase inhibitor preserved coronary arteriolar dilations to serotonin and adenosine (Figure 6) but failed to block vasoconstriction in response to PKC activation (Figure 1B). These results indicate that Rho kinase is not involved in the vasoconstriction evoked by PKC but mediates the signaling to oxidative stress and endothelial dysfunction. The activation of PKCβ2, as suggested by the present study, appears to promote vascular superoxide production from xanthine oxidase through Rho kinase and JNK activations (Figure 7). It is noted that both Rho kinase and JNK inhibitors exhibited the same efficacy in preserving vasomotor function (Figure 6), suggesting their action on the same signaling pathway. Although the signaling sequence between Rho kinase and JNK in the present study remains unclear, it has been shown that JNK activity can be regulated by Rho kinase in various cell types, including vascular cells [72,73]. Recently, the activation of JNK by Rho kinase has been suggested to mediate enhanced venular constriction to endothelin-1 in diabetic animals [66]. The sequential activation of Rho kinase and JNK for endothelial dysfunction following PKCβ2 activation deserves further investigation. 

In summary, the present study demonstrated that activation of PKC by PDBu elicits at least two different pathways, one leads to Rho kinase-independent vasoconstriction, and the other leads to Rho kinase-dependent superoxide production and impairment of endothelium-dependent NO-mediated dilation in porcine coronary arterioles. Selective activation of PKCβ2, by a sub-vasomotor concentration of PDBu, appears to trigger JNK-dependent activation of xanthine oxidase for superoxide production via Rho kinase signaling (Figure 7). It appears that elevated basal production of superoxide without activation of smooth muscle contractile machinery is sufficient to evoke endothelial dysfunction. Because PKC is an important regulator for vascular smooth muscle function and a pathological target in vascular disorders [74], understanding the direct role of PKC activation in the intact vessel could advance the design of therapeutic tools for disease prevention and treatment.

## 4. Materials and Methods

### 4.1. Materials

The animal procedures and protocols were carried out under the guidance of the Animal Care and Use Committee (ID: 2007-008-R approved on February 16, 2010) at the Texas A&M University Health Science Center and Baylor Scott & White Health (Temple, TX, USA). Pigs (8–12 weeks old, either sex) were purchased from Real Farms (San Antonio, TX, USA) and sedated with Telazol (4–8 mg/kg, intramuscularly), anesthetized with 2–5% isoflurane (Baxter Healthcare Co., Deerfield, IL, USA), heparinized with heparin (1000 U/kg, intravenously via marginal ear vein; Cardinal Health, Dublin, OH, USA), and intubated. A left lateral thoracotomy was performed, and the heart was swiftly removed and immersed in cold (5 °C) saline solution as previously described [75,76].

### 4.2. Isolation and Cannulation of Coronary Microvessels

To eliminate the confounding influences from hemodynamic, neurohumoral, and myocardial metabolic changes, coronary arterioles were isolated and studied ex vivo [77]. Subepicardial coronary arterioles (40–80 μm internal diameter in situ) were carefully dissected out and cannulated with a pair of glass micropipettes in a vessel chamber containing physiological salt solution (PSS) and 1% bovine serum albumin (Thermo Fisher Scientific, Bridgewater, NJ, USA) [49]. The isolated vessels were pressurized to 60 cmH_2_O intraluminal pressure, based on the reported level of pressure distribution in vivo [78], by two independent reservoirs [77]. The vessels developed a basal tone within 40 min at a 37 °C bath temperature. The internal diameters of the arterioles were recorded throughout the experiment using videomicroscopic techniques [49].

### 4.3. Effect of PDBu on Vasodilator Function of Isolated Coronary Arterioles

After development of a stable resting diameter (i.e., basal tone), the concentration-dependent vasoconstriction of coronary arterioles to PDBu was constructed and the involvements of PKC subtypes and Rho kinase were assessed with inhibitor Gö6983 (1 µmol/L) [79] against a broad-spectrum of PKC (PKCα, PKCβ, PKCγ, PKCδ and PKCζ), CGP53353 (0.3 µmol/L) against PKCβ2 [42,80], and Y27632 (0.1 µmol/L; Calbiochem, San Diego, CA, USA) against Rho kinase [20]. In another series of experiments, coronary arterioles were exposed to a sub-vasomotor level of PDBu (1 nmol/L) for 60 min and the vessels were challenged with the endothelium-dependent, NO-mediated vasodilators serotonin (0.1 nmol/L to 0.1 μmol/L) and adenosine (0.1 nmol/L to 10 μmol/L) and the endothelium-independent vasodilator sodium nitroprusside (0.1 nmol/L to 10 µmol/L). To examine the contribution of superoxide and hydrogen peroxide to the sub-vasomotor effect of PDBu, the vasodilations to serotonin and adenosine were assessed before and after co-incubation of PDBu with superoxide scavenger 4-hydroxy-2,2,6,6-tetramethylpiperidine-1-oxyl (Tempol, 1 mmol/L) [55] and hydrogen peroxide scavenger PEG-catalase (500 U/mL) [81], respectively. The roles of NAD(P)H oxidase and xanthine oxidase were determined in the presence of their respective inhibitors apocynin (100 µmol/L) and allopurinol (10 μmol/L) [20]. In another series of studies, the involved PKC subtypes, NO, and stress-activated protein kinases were examined by co-treating the vessels with a sub-vasomotor concentration of PDBu and inhibitors of pan-PKC (Gö6983, 1 µmol/L), PKCβ2 (CGP53353, 0.3 µmol/L), NOS (L-NAME, 10 µmol/L), JNK (SP600125, 5 µmol/L; Calbiochem), p38 kinase (SB203580, 0.1 µmol/L; Calbiochem), or Rho kinase (Y27632, 0.1 µmol/L). The concentrations of inhibitors used in the present study have been shown to be effective in microvessel preparations [20,22,55,79,81,82]. In each pharmacological intervention, 4 to 6 arterioles were used. All drugs, unless otherwise stated, were obtained from Sigma-Aldrich (St. Louis, MO, USA).

### 4.4. Immunohistochemical Detection of eNOS and PKCβ2

Coronary arterioles were embedded in OCT compound (Tissue-Tek; Electron Microscopy Sciences, Hatfield, PA, USA) and frozen sections (10 μm thickness) were fixed in 4% paraformaldehyde for immunohistochemical analysis as described previously [83,84]. Immunolabelling was performed using a mouse monoclonal antibody against eNOS (610297, 1:100 dilution; BD Biosciences, Franklin Lakes, NJ, USA) and a rabbit polyclonal antibody against PKCβ2 (sc-210, 1:100 dilution; Santa Cruz Biotechnology, Dallas, TX, USA). The slides were then incubated with rhodamine red-labeled (Jackson Laboratories, West Grove, PA, USA) and FITC-labeled (Jackson Laboratories) secondary antibodies. Staining control tissues were exposed for the same duration to non-immune serum (Jackson Laboratories) in place of primary antibody. Slides were observed for red (rhodamine red for PKCβ2) and green (FITC for eNOS) images under a fluorescence microscope (Axiovert 200, Zeiss, Jena, Germany) and analyzed using ImageJ software (National Institutes of Health, Bethesda, MD, USA) as described previously [20].

### 4.5. Detection of Vascular Superoxide 

Superoxide production in isolated coronary arterioles was evaluated with the fluorescent dye dihydroethidium (DHE; Polysciences, Warrington, PA, USA) as described previously [20]. Isolated coronary arterioles, 40 to 100 μm in diameter and 1.5 mm in length, were incubated with a vehicle solution, PDBu (1 nmol/L), or PDBu plus Tempol (1 mmol/L), and then stained with DHE (4 µmol/L) for 30 min. After being washed, arterioles were embedded in OCT compound (Tissue-Tek) for frozen section (10 μm thickness). The DHE fluorescence image was taken at excitation/emission wavelength of 360/460 nm with a fluorescence microscope (Axiovert 200, Zeiss). Control and experimental tissues were set on the same slide and processed and analyzed under the same conditions.

### 4.6. Data Analysis

Vasoconstriction to PDBu was normalized to the resting vessel diameter following development of vascular tone [20]. Arteriolar responses to vasodilators were normalized to the maximal diameter obtained in the Ca^2+^-free solution containing 0.1 mmol/L sodium nitroprusside and are expressed as a percentage of the maximal dilation [51]. Data are reported as the mean ± SEM, and “*n*” represents the number of vessels (1–2 vessels per pig). The changes in resting tone by PDBu and pharmacological inhibitors were analyzed with one-way analysis of variance (ANOVA) followed by a Dunnett’s multiple comparison test. The fluorescence images from DHE staining were quantified using ImageJ software as previously described [22] and then analyzed with a Student’s *t*-test. The significance of the experimental interventions on vasomotor responses to serotonin and adenosine was analyzed with a two-way ANOVA followed by Tukey’s multiple comparison test. A value of *p* < 0.05 was considered statistically significant.

## Data Availability

The datasets generated and/or analyzed for the current study are available from the corresponding authors upon reasonable request.

## Supplementary Materials

The following are available online at https://www.mdpi.com/article/10.3390/ijms22189763/s1.
