# Supplementary material for: Activation of Coronary Arteriolar PKCβ2 Impairs Endothelial NO-Mediated Vasodilation: Role of JNK/Rho Kinase Signaling and Xanthine Oxidase Activation
Source: Int J Mol Sci. 2021 Sep 9;22(18):9763. doi: 10.3390/ijms22189763 (PMC8471475; doi:10.3390/ijms22189763)
Supplement: Supplementary file 1 [file ijms-22-09763-s001.zip › ijms-1369027-supplementary.pdf]

Figure S1: Effect of PDBu on vasodilation to sodium nitroprusside.

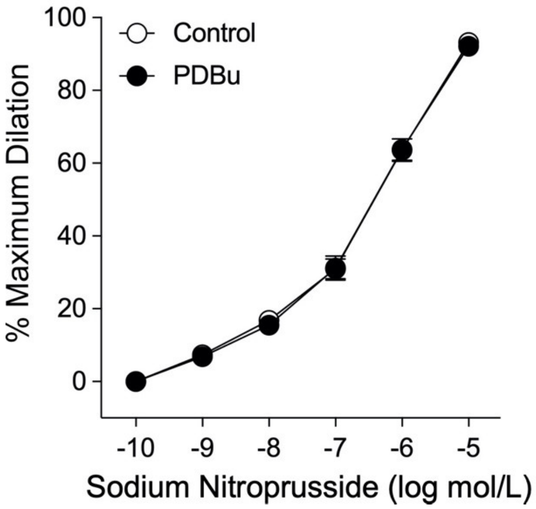

Figure S1. The dilation of coronary arterioles in response to sodium nitroprusside was examined before and after incubation with 1 nmol/L PDBu for 60 minutes. The vasodilator response to sodium nitroprusside was not altered by PDBu (n=5, two-way repeated measures ANOVA). n = number of vessels.
